# Constrained Amorphous Interphase and Mechanical Properties of Poly(3-Hydroxybutyrate-*co*-3-Hydroxyvalerate)

**DOI:** 10.3389/fchem.2019.00790

**Published:** 2019-11-19

**Authors:** Maria Cristina Righetti, Laura Aliotta, Norma Mallegni, Massimo Gazzano, Elisa Passaglia, Patrizia Cinelli, Andrea Lazzeri

**Affiliations:** ^1^CNR-IPCF, National Research Council-Institute for Chemical and Physical Processes, Pisa, Italy; ^2^Department of Civil and Industrial Engineering, University of Pisa, Pisa, Italy; ^3^CNR-ISOF, National Research Council-Institute of Organic Synthesis and Photoreactivity, Bologna, Italy; ^4^CNR-ICCOM, National Research Council-Institute for the Chemistry of OrganoMetallic Compounds, Pisa, Italy

**Keywords:** crystallinity, mobile amorphous fraction, rigid amorphous fraction, mechanical properties, elastic modulus

## Abstract

In the present study, for the first time the evolution of tensile mechanical properties of different poly(3-hydroxybutyrate-*co*-3-hydroxyvalerate) copolymers (PHBV8 and PHBV12, with 8 mol% and 12 mol% of HV co-units, respectively) as a function of the storage time at room temperature has been investigated in parallel with the quantification of the crystalline, mobile amorphous, and rigid amorphous fractions. A comparison with the evolution of the crystalline and amorphous fractions in the homopolymer poly(3-hydroxybutyrate) (PHB) was also performed. For all the samples, the crystallinity was found to slightly increase during storage. In parallel, the mobile amorphous fraction (MAF) decreased markedly, with the result that a relevant increase in the rigid amorphous fraction (RAF) was detected. The RAF content in the copolymers was lower than that of PHB. For all the samples, the RAF formation during aging was ascribed to the growth of secondary crystals in geometrically restricted areas. It was demonstrated that the storage at *T*_*room*_ leads in PHB, PHBV8, and PHBV12 to a progressive increase in the total solid fraction (crystal phase + rigid amorphous fraction) and to a simultaneous physical aging of the rigid amorphous fraction. The two different processes cannot be separated and distinguished, so that only the resulting effect on the mechanical properties was considered. The experimental elastic modulus of both PHBV8 and PHBV12 was found to increase regularly with the total solid fraction, as well as the tensile strength. Conversely, the elongation at break turned out to be an increasing function of the mobile amorphous fraction. The elastic moduli of the crystalline, mobile amorphous, and rigid amorphous fractions of PHBV8 and PHBV12 were estimated by means of a three-phase modified Takayanagi's model, to take into account also the contribution of the rigid amorphous fraction. The calculated values were found in agreement with theoretical expectations.

## Introduction

Poly(3-hydroxybutyrate) (PHB) is the most widespread and best characterized homopolymer of the polyhydroxyalkanoate (PHA) family. PHAs are a group of biocompatible and completely biodegradable polyesters, synthesized by many bacteria as intracellular carbon and energy reserve (Sudesh et al., 2000). Owing to their biodegradability in compost, soil and marine water, they can be utilized for many different applications, in particular for single use packaging and in agriculture (Bugnicourt et al., 2014). PHB is a semi-crystalline isotactic stereo-regular polymer with 100% (*R*)-configuration. The as-received PHB, isolated from bacteria, is a high crystallinity powder, with average crystallinity of ~65% (Sudesh et al., 2000), whereas the *in vivo* PHB is an amorphous polymer (Kawaguchi and Doi, 1990).

The thermal properties (glass transition temperature and melting temperature) of PHB, as well as the elastic modulus and tensile strength are close to those of polypropylene (Sudesh et al., 2000). Conversely, its elongation at break is noticeably lower, which makes PHB a material much more brittle than polypropylene. This poor mechanical property was attributed to the large spherulites that grow as a consequence of a low nucleation density (Barham, 1984). These spherulites generally show radial and circumferential cracks, from which fractures can originate and propagate (Barham and Keller, 1986). In addition, it has been widely reported that PHB, after initial crystallization, undergoes embrittlement during storage at room temperature (de Koning and Lemstra, 1993). This embrittlement was ascribed to further secondary crystallization that occurs during storage at room temperature, being the glass transition temperature of PHB close to 0°C. The further and progressive crystallization was found also to seriously constrain the amorphous phase, thus further promoting the PHB embrittlement (de Koning and Lemstra, 1993).

A detailed quantitative thermal analysis of the evolution of the crystalline and amorphous fractions of PHB upon storage at room temperature was performed after crystallization at different cooling rates (Di Lorenzo and Righetti, 2013a,b). The permanence at room temperature was found to induce progressive growth of PHB crystals, and simultaneous vitrification of constrained amorphous segments in proximity of the secondary crystalline regions. As the length of the polymer molecules is much higher than the dimensions of the crystalline phase, at least in one direction, the decoupling between crystalline and amorphous phases is generally partial, and there exists an interphase, between the crystalline and amorphous regions, made of amorphous chain portions with mobility delayed by the near crystalline structures. This nanosized constrained amorphous interphase, named rigid amorphous fraction (RAF), vitrifies and devitrifies at temperatures higher than the bulk *T*_*g*_, in a wide temperature range, because various degrees of coupling exist between the non-crystallized and the crystallized chain segments (Wunderlich, 2003; Righetti et al., 2014, 2016). Prolonged storage at room temperature of PHB causes increase in both crystallinity (final crystallinity: 70–80%) and RAF (final RAF: 20–25%), so that the overall solid fraction (crystals + RAF) becomes very close to the totality of the material. An influence of the stiff RAF on the material's mechanical response is expected when the amorphous and crystalline phases are largely coupled, as occurs in PHB. Thus, also the constrained amorphous fraction was supposed to partially contribute to the worse ductility that PHB exhibits after prolonged storage at room temperature.

The establishment of a nanosized rigid amorphous fraction in PHB was found to depend on the temperature at which crystallization occurs (Righetti et al., 2013). RAF grows simultaneously with the crystal phase during crystallization at 30°C, whereas RAF starts to develop during the final stages of the crystallization process if crystallization occurs at higher temperatures. The temperature of 70°C was supposed to be the limit for the presence of RAF in PHB.

Another phenomenon that can influence the properties of PHB during storage at room temperature is the physical aging of the rigid amorphous fraction. It was proven that in PHB the immobilized RAF fraction can undergo structural relaxation during storage at *T*_*room*_ (Di Lorenzo et al., 2012), even though a quantitative and systematic study on the RAF structural relaxation has never been performed. In a quite recent study on PHB, the progressive increase in the elastic modulus and the decrease in the elongation at break at *T*_*room*_ were ascribed to the RAF physical aging, although a concomitant quantification of the three-phase structure was not accomplished (Srubar et al., 2012). Thus, two different processes occur in PHB during storage at *T*_*room*_: progressive increase in the total solid fraction (crystals + RAF) and concomitant structural relaxation of the RAF.

The intrinsic brittleness, together with the low resistance to thermal degradation during processing (Kunioka and Doi, 1990) has restricted up to now the practical applications of PHB. One approach widely applied to improve the mechanical properties of PHB is the incorporation of different hydroxyalkanoate monomeric co-units in the PHB chain, through microbial synthesis. Many different PHAs have been produced and characterized in a wide range of physical properties. The most common and studied random copolymers of PHB are poly(3-hydroxybutyrate-*co*-3-hydroxyvalerate) (PHBV). These copolymers exhibit lower stiffness and brittleness, higher elongation at break and increased tensile strength with respect to PHB (Laycock et al., 2014). These improved mechanical properties have been attributed to the smaller dimensions of the crystallites, as the hydroxyvalerate (HV) co-units act as defects in the PHB lattice.

It is well-known that PHBV copolymers exhibit isodimorphism, i.e., cocrystallization of both the co-monomers in the crystal lattices of the two homopolymers PHB and poly(3-hydroxyvalerate) (PHV), depending on the copolymer composition (Bluhm et al., 1986; Kuniota et al., 1989; Scandola et al., 1992). The crystalline phase of the copolymers PHBV transforms from the PHB lattice to the PHV lattice at a composition of about 50 mol% (Kuniota et al., 1989). The unit cell of both PHB and PHV, grown under the typical conditions of melt and cold crystallization, is orthorhombic, and also the chain conformation and packing are similar, except for a decrease of nearly 10% in the *c*-axis of the HV unit cell (Sudesh and Abe, 2010). The incorporation of the HV units in the PHB cell perturbs the lattices dimensions, which progressively increase, due to steric effects, while the incorporation of the HB units in the PHV lattice has no apparent effect on the crystallographic parameters (Nakamura et al., 1992; Scandola et al., 1992). The less bulky component co-crystallizes more easily than the bulkier one, so that more HB co-units crystallize in the PHV lattice than HV in the PHB lattice (Kamiya et al., 1991). The result is that the composition of the crystalline phase of PHBV is different from the overall composition: for HV content lower than about 40 mol%, the actual HV percentage in the crystalline regions is approximately half or less of nominal amount (Kamiya et al., 1991; Bonthrone et al., 1992).

The RAF quantification in PHBV copolymers has been presented in few studies (Chen et al., 2002; Cheng and Sun, 2009; Cheng et al., 2009; Esposito et al., 2016). An analysis performed by solid-state NMR on the structure and mobility of the non-crystalline region of PHB and PHBV revealed that a rigid component of the amorphous phase is present also in the PHBV copolymers (Chen et al., 2002). The RAF content, which decreases with increasing the temperature (from 20 to 80°C), is lower in the PHBV copolymers with respect to PHB, and progressively decreases with increasing the HV content (from 4 to 10 mol%). From positron annihilation lifetime spectroscopy (Cheng and Sun, 2009; Cheng et al., 2009), the presence of a rigid amorphous fraction in PHBV (5 mol% of HV co-units) was attested. This constrained amorphous fraction was found to devitrify in the temperature range between 40 and 80°C.

Also for PHBV copolymers, the mechanical properties as a function of the storage time at *T*_*room*_ were measured, although without a simultaneous quantification of the three-phase structure (El-Hadi et al., 2002; Srubar et al., 2012). As for PHB, with increasing the aging time, the elastic modulus increases, whereas the elongation at break decreases.

For semicrystalline polymers, generally only the effect of the crystalline parameters (for example crystallinity, morphology and lamellar thickness) on the mechanical properties is investigated (Basset et al., 1999; Doyle, 2000; Fatahi et al., 2007; Stern et al., 2007; Huang et al., 2011; Auclerc et al., 2019). Actually, in addition to the crystal fraction and morphology, also the ratio between RAF and MAF should largely affect the properties of semi-crystalline polymers (Rastogi et al., 2004). It was suggested that the mechanical characteristics of the RAF in the glassy state might be similar to those of the crystal phase (Zia et al., 2008). For poly(1-butene) a linear relationship between the elastic modulus at room temperature and the total solid content (crystal + RAF) led to suppose that the elastic modulus of the RAF is comparable to that of the crystal phase (Di Lorenzo and Righetti, 2008). For polyamide 6, a strong increase in the storage modulus at low crystalline fraction was explained as due to additional RAF formation (Kolesov and Androsch, 2012). A deconvolution analysis of the viscoelastic properties of PET allowed to quantify separately the mechanical properties of the mobile and rigid amorphous fractions. At 90°C, a higher modulus was derived for the RAF with respect to that of the mobile amorphous fraction (MAF) (Nguyen et al., 2015). For poly(l-lactic acid), an increase in the elastic moduli and a parallel decrease in ductility were found in samples with increasing crystalline and rigid amorphous fractions (Lizundia et al., 2013), whereas the higher fragility and smaller ductility of recycled PET was explained by considering that the RAF progressively increases with the processing cycles (Badia et al., 2012).

In the present study, for the first time the evolution of tensile mechanical properties of PHBV copolymers as a function of the storage time at *T*_*room*_ has been investigated in connection with the quantification of the crystalline and rigid amorphous fractions.

In addition, the elastic modulus of the PHBV copolymers has been theoretically described by expanding the classical two-phase Takayanagi's model (Takayanagi et al., 1964, 1967) to a three-phase system, in order to take into account and possibly quantify the contribution of the rigid amorphous fraction.

## Materials and Methods

### Chemicals

Poly[(R)-3-hydroxybutyrate] (PHB) and poly(3-hydroxybutyrate-*co*-3-hydroxyvalerate) with 8 and 12 mol% of HV co-units (PHBV8 and PHBV12, respectively) were purchased from Sigma Aldrich S.R.L (Milan, Italy).

### Characterization

#### Molar Mass Characterization

Number-average molar mass (*M*_*n*_) and weight-average molar mass (*M*_*w*_) of the as-received PHB, PHBV8, and PHBV12 samples were determined using size exclusion chromatography (SEC), Agilent Technologies 1200 Series (Santa Clara, CA, USA) and calculated with the Agilent ChemStation Software. The instrument was equipped with an Agilent degasser, an isocratic HPLC pump, two PLgel 5 μm MiniMIX-D columns conditioned at 35°C and an Agilent refractive index (RI) detector. Chloroform (CHCl_3_) was used as mobile phase at a flow rate of 0.3 mL/min. The system was calibrated with polystyrene standards in a range from 500 to 3 × 10^5^ g/mol. Samples were dissolved in CHCl_3_ (2 mg/mL) and filtered through a 0.20 μm syringe filter before the analysis.

#### Sample Preparation for Mechanical and Thermal Characterization

Before processing, the as-received PHB, PHBV8, and PHBV12 samples were dried at 60°C for 24 h. The PHB, PHBV8, and PHBV12 samples were processed by using a MiniLab II HAAKE Rheomex CTW 5 (Waltham, MA, USA), a co-rotating conical twin-screw extruder. The molten materials were transferred from the mini extruder through a preheated cylinder to a mini injection molder (Thermo Scientific HAAKE MiniJet II) (Waltham, MA, USA), which allows to prepare dog-bone tensile bars specimens, to be used for thermal and mechanical characterization. The dimensions of the dog-bone tensile bars Haake 3 type were: width in the larger section: 10 mm, width in the narrow section: 4.8 mm, thickness 1.35 mm, and length 90 mm. The extruder operating conditions adopted for the samples are reported in Table 1.

**Table 1:** Operating conditions used for the extrusion and injection molding processes of PHB, PHBV8, and PHBV12.

	**Extrusion temperature (°C)**	**Screw speed (rpm)**	**Cycle time (s)**	**Injection temperature (°C)**	**Injection pressure (bar)**	**Molding time (min)**	**Mold temperature (°C)**
PHB	185	100	90	185	150	4	80
PHBV8	175	100	90	175	150	4	80
PHBV12	175	100	90	175	150	4	80

The PHB, PHBV8, and PHBV12 samples processed at 80°C for 4 min were stored at room temperature (*T*_*room*_≅ 25°C) and the thermal and mechanical properties analyzed after different storage times (*t*_*a*_), ranging from 1 to 70 days.

#### Thermal Characterization by Differential Scanning Calorimetry (DSC) and Temperature-Modulated Differential Scanning Calorimetry (TMDSC)

Differential scanning calorimetry (DSC) and temperature-modulated calorimetry (TMDSC) measurements were performed with a Perkin Elmer Differential Scanning Calorimeter DSC 8500 equipped with an IntraCooler III as refrigerating system. The instrument was calibrated in temperature with high purity standards (indium, naphthalene, cyclohexane) according to the procedure for standard DSC (Sarge et al., 1997). Energy calibration was performed with indium. Dry nitrogen was used as purge gas at a rate of 30 mL/min.

As preliminary investigation, the thermodynamic specific heat capacities in the solid and liquid state ($c_{p}^{s}$ and $c_{p}^{l}$, respectively) of PHV, PHBV8, and PHBV12 were measured. The homopolymer PHB was heated to 190°C, and maintained at this temperature for 3 min, whereas PHBV8 and PHBV12 were treated at 180°C for 3 min, in order to completely destroy all traces of previous crystalline order. After melting, the PHB, PHBV8, and PHBV12 samples were quickly removed from the DSC apparatus, and quenched in liquid nitrogen, to obtain completely amorphous samples. The apparent specific heat capacities (*c*_*p,app*_) of the quenched PHB, PHBV8, and PHBV12 were measured at the heating rate of 10 K/min from −80 to 190°C.

The PHB, PHBV8, and PHBV12 samples processed at 80°C for 4 min and stored at room temperature (*T*_*room*_ ≅ 25°C) for different *t*_*a*_s, ranging from 1 to 70 days, were analyzed by DSC. The *c*_*p,app*_ curves were obtained at 10 K/min, from −50°C (PHB) or −70°C (PHBV8 and PHBV12) up to complete fusion.

Temperature modulated differential scanning (TMDSC) analysis was also carried out on PHB, PHBV8, and PHBV12 samples after storage of 20 months at *T*_*room*_. The measurements were performed by using a saw-tooth modulation temperature program, with a temperature amplitude (*A*_*T*_) of 0.5 K, a modulation period (*p*) of 120 s, and an average heating rate of 2 K/min. According to the mathematical treatment of TMDSC data, the modulated temperature and the heat flow rate curves can be approximated to Fourier series (Wunderlich, 1997; Wurm et al., 1998; Androsch et al., 2000). From the ratio between the amplitudes of the first harmonic of the heat flow rate and temperature, the reversing specific heat capacity (*c*_*p,rev*_) is obtained:

(1) $$\begin{array}{l} c_{p,rev}\left(\omega ,t\right)=\frac{A_{HF}\left(t\right)}{A_{T}\left(t\right)}\frac{K\left(\omega ,t\right)}{m\;\omega } \end{array}$$

where ω is the frequency of temperature modulation (ω = 2π/*p*), *t* the time, *A*_*HF*_(*t*), and *A*_*T*_(*t*) the amplitudes of the first harmonics of the heat flow rate and temperature modulation, respectively, and *m* the mass of the sample. The frequency-dependent calibration factor, *K*(ω,*t*), determined by calibration with sapphire, was 1.00 for *p* = 120 s. The *c*_*p,rev*_ curves were compared with the *c*_*p,app*_ curves obtained for the same samples at 10 K/min.

#### Tensile Characterization

Tensile tests on the PHBV8 and PHBV12 samples processed at 80°C for 4 min were performed after different *t*_*a*_s at *T*_*room*_ (1–60 days) at a crosshead speed of 10 mm/min, by means of an INSTRON 5500 R universal testing machine (Canton MA, USA), equipped with a 10 kN load cell and interfaced with a computer running the Testworks 4.0 software (MTS Systems Corporation, Eden Prairie MN, USA). At least five specimens were tested for each sample in according to the ASTM D638.

#### XRD Analysis

XRD patterns of PHB, PHBV8, and PHBV12 samples processed at 80°C for 4 min and stored at *T*_*room*_ for different *t*_*a*_s (1–70 days) were collected using a PANalytical X'PertPro diffractometer equipped with a fast solid state X'Celerator detector and a Cu X-ray tube producing X-rays with a wavelength of 0.15418 nm. The X-ray crystallinity was calculated as the ratio of the areas of the crystalline peaks and the total area of the background-corrected diffraction profile. The lattice constants were calculated from the positions of the 8–10 most intense reflections, by least-squares refinements.

## Results and Discussion

### Molar Mass Characterization of PHB, PHV8, and PHBV12

The number-average molar mass (*M*_*n*_) and the mass-average molar mass (*M*_*w*_) values of the as-received PHB, PHBV8, and PHBV12 samples, measured by SEC, are listed in Table 2. The table shows that the molar masses of the copolymers PHV8 and PHBV12 are similar, whereas *M*_*n*_ and *M*_*w*_ of PHB are markedly higher. This prevents a direct comparison of the thermal behavior of the copolymers with that of the homopolymer, which will be reported only for a qualitative paralleling.

**Table 2:** Number-average molar mass (*M*_*n*_) and mass-average molar mass (*M*_*w*_) of the as-received PHB, PHBV8, and PHBV12 samples (estimated error: ±10,000 g/mol).

	***M_**n**_* (g/mol)**	***M_**w**_* (g/mol)**
PHB	440,000	610,000
PHBV8	110,000	210,000
PHBV12	120,000	220,000

### Thermodynamic Specific Heat Capacities of PHB, PHBV8, and PHBV12

**Figure 1:**
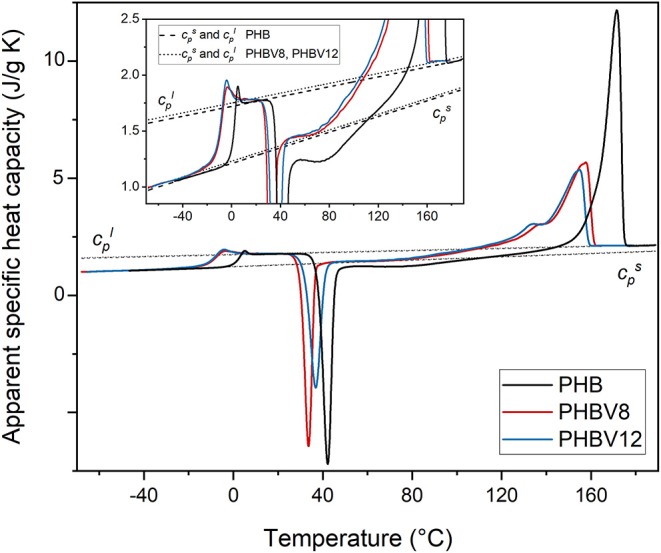
Apparent specific heat capacity (*c*_*p,app*_) of PHB, PHBV8, and PHBV12 after quench from melt, measured at 10 K/min. The dashed and dotted lines are the thermodynamic solid and liquid specific heat capacities of PHB and the copolymers PHBV8 and PHBV12 ($c_{p}^{s}$ and $c_{p}^{l}$, respectively). The inset is an enlargement of the *c*_*p,app*_ curves in the *T*_*g*_ and cold crystallization regions.

For an accurate determination of the crystalline, mobile amorphous and rigid amorphous weight fractions of the samples, as preliminary analysis, the thermodynamic solid and liquid specific heat capacities ($c_{p}^{s}$ and $c_{p}^{l}$, respectively) of PHB, PHBV8, and PHBV12 were determined. The apparent specific heat capacities (*c*_*p,app*_) of initially amorphous PHB, PHBV8, and PHBV12 samples were measured on heating at 10 K/min, as shown in Figure 1. Both the thermodynamic specific heat capacity and the latent heat absorbed or released during phase transitions contribute to *c*_*p,app*_, with the result that *c*_*p,app*_ corresponds to the thermodynamic specific heat capacity only outside the phase transition regions (Wunderlich, 2003, 2008).

As expected, the figure shows that the glass transition of the copolymers PHBV8 and PHBV12 is located at lower temperatures with respect to PHB (Laycock et al., 2014), and that *T*_*g*_ of PHBV8 is only slightly higher than that of PHBV12 (PHB: *T*_*g*_ = 1.5°C; PHBV8: *T*_*g*_ = −9°C; PHBV12: *T*_*g*_ = −10°C). The $c_{p}^{l}$ lines were constructed by connecting the region above the glass transition with the melt, whereas the $c_{p}^{s}$ trends were obtained by extrapolation of the *c*_*p,app*_ data below the glass transition. The resulting $c_{p}^{s}$ and $c_{p}^{l}$ expressions are reported in Table 3. For PHB, the measured thermodynamic solid and liquid specific heat capacities are in agreement with literature data within ± 2% (Schick et al., 2001; Czerniescka et al., 2014). To the best of our knowledge, thermodynamic solid and liquid specific heat capacities of PHBV8 and PHBV12 have never been reported.

**Table 3:** Thermodynamic solid and liquid specific heat capacities ($c_{p}^{s}$ and $c_{p}^{l}$, respectively) of PHB, PHBV8, and PHBV12 (with *T* in °C) (estimated error: ± 0.03 J/g K).

	$c_{p}^{s}$ **(J/g K)**	$c_{p}^{l}$ **(J/g K)**
PHB	1.21 + 0.0035 *T*	1.72 + 0.0022 *T*
PHBV8	1.23 + 0.0035 *T*	1.75 + 0.0022 *T*
PHBV12	1.23 + 0.0035 *T*	1.75 + 0.0022 *T*

Figure 1 and Table 3 show that the increment of the specific heat capacity at *T*_*g*_ (Δ*c*_*p*_) for the copolymers is slightly higher than that of PHB, which can be connected with the higher chain mobility of the copolymers and the associated higher degrees of freedom that become active at the respective *T*_*g*_s.

At temperatures higher than *T*_*g*_, all the amorphous samples undergo an intense cold crystallization process. As the HV co-units act as defects for the PHB crystallization (Nakamura et al., 1992; Scandola et al., 1992), the cold crystallization of PHBV12 occurs at slightly higher temperature with respect to PHV8. Due to its high molar mass, cold crystallization of PHB is observed at temperatures higher than that of the copolymers, which makes impossible a quantitative comparison of the cold crystallization kinetics of the different samples.

More importantly, both PHB and the copolymers exhibit a double exotherm in the cold crystallization region, which indicates that this event is a two-stage process. The phenomenon was widely discussed for PHB (Di Lorenzo et al., 2012) and correlated with the role of vitrification/devitrification of the rigid amorphous fraction on the cold crystallization kinetics. During the first stage of the cold crystallization, which occurs at quite low temperatures, RAF develops simultaneously with the crystals growth, due to the low mobility of the polymer chains. This coupling between the crystalline and amorphous segments, which creates an immobilized amorphous layer at the crystals surfaces, progressively hinders further crystallization, but additional increase in temperature is able to produce the RAF mobilization. When the amorphous layers connected to the crystals have reached sufficient mobility, crystallization can proceed, because chain rearrangements at the crystal/amorphous interphase become possible. This second stage, connected to the exotherm at higher temperatures, can be approximately associated with the temperature at which full mobilization of the RAF is attained, which for PHB is around 70°C (Di Lorenzo et al., 2012; Righetti et al., 2013).

The double exotherm present in the *c*_*p,app*_ curves of PHBV8 and PHBV12 attests that RAF develops also in PHBV8 and PHBV12, and that the temperature limit for the presence of this constrained amorphous interphase in the copolymers is lower with respect to PHB. A reduced devitrification temperature of the RAF in the copolymers might be connected to the respective lower molar mass, although it sounds more likely that it has to be associated with the higher chain mobility that characterizes the HV co-units.

As expected for copolymers, the melting temperature of PHBV8 and PHBV12 appears lower than that of the homopolymer, due to the disturbance of crystal packing by partial inclusion of HV co-units (Bluhm et al., 1986).

### Apparent Specific Heat Capacities of PHB, PHBV8, and PHBV12 During Aging at *T_room_*

**Figure 2:**
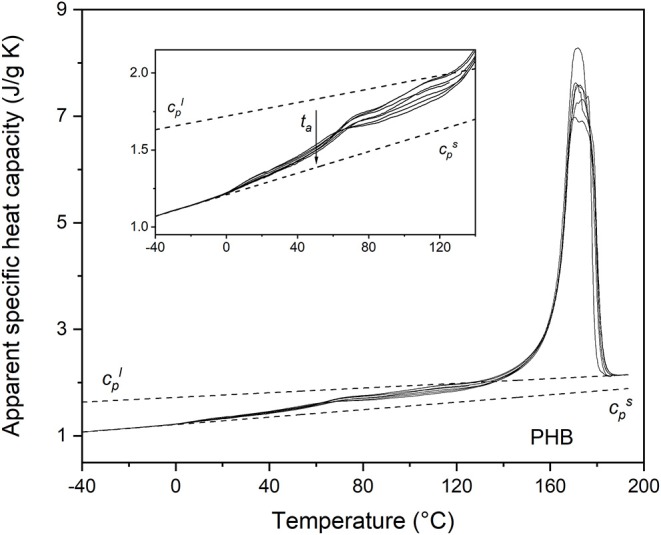
Apparent specific heat capacity (*c*_*p,app*_) of PHB after crystallization at *T*_*c*_ = 80°C for 4 min and storage at *T*_*room*_ for increasing *t*_*a*_s (1 ÷ 70 days), measured at 10 K/min. The dotted lines are the thermodynamic solid and liquid specific heat capacities ($c_{p}^{s}$ and $c_{p}^{l}$, respectively). The inset is an enlargement of the *c*_*p,app*_ curves in the *T*_*g*_ region.

**Figure 3:**
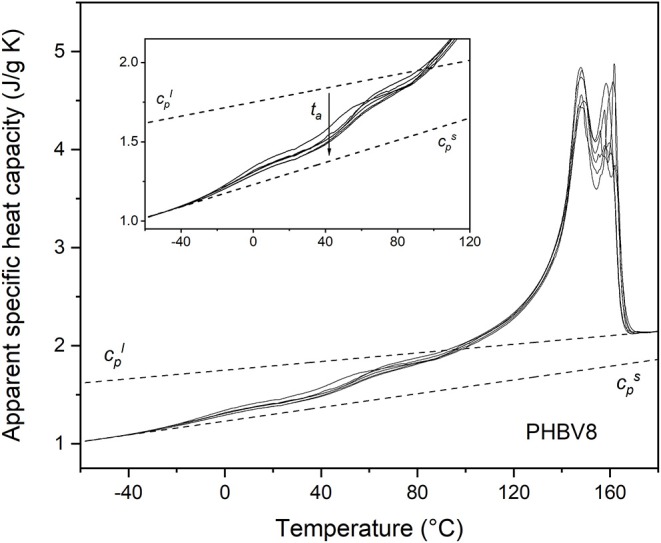
Apparent specific heat capacity (*c*_*p,app*_) of PHBV8 after crystallization at *T*_*c*_ = 80°C for 4 min and storage at *T*_*room*_ for increasing *t*_*a*_s (1 ÷ 70 days), measured at 10 K/min. The dotted lines are the thermodynamic solid and liquid specific heat capacities ($c_{p}^{s}$ and $c_{p}^{l}$, respectively). The inset is an enlargement of the *c*_*p,app*_ curves in the *T*_*g*_ region.

**Figure 4:**
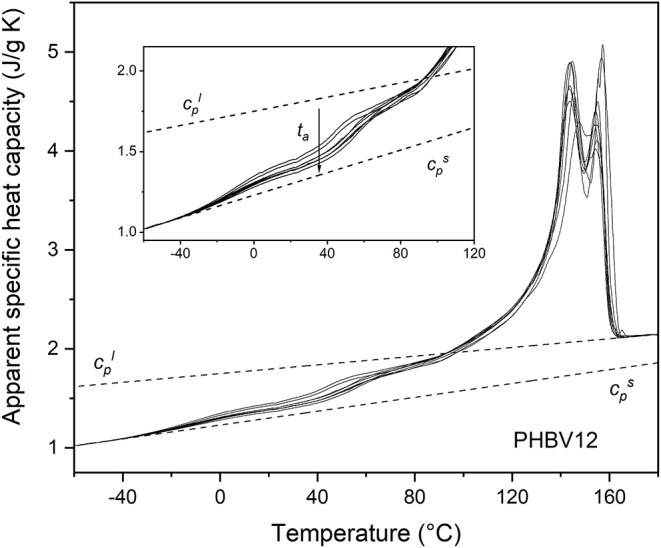
Apparent specific heat capacity (*c*_*p,app*_) of PHBV12 after crystallization at *T*_*c*_ = 80°C for 4 min and storage at *T*_*room*_ for increasing *t*_*a*_s (1 ÷ 70 days), measured at 10 K/min. The dotted lines are the thermodynamic solid and liquid specific heat capacities ($c_{p}^{s}$ and $c_{p}^{l}$, respectively). The inset is an enlargement of the *c*_*p,app*_ curves in the *T*_*g*_ region.

**Figure 5:**
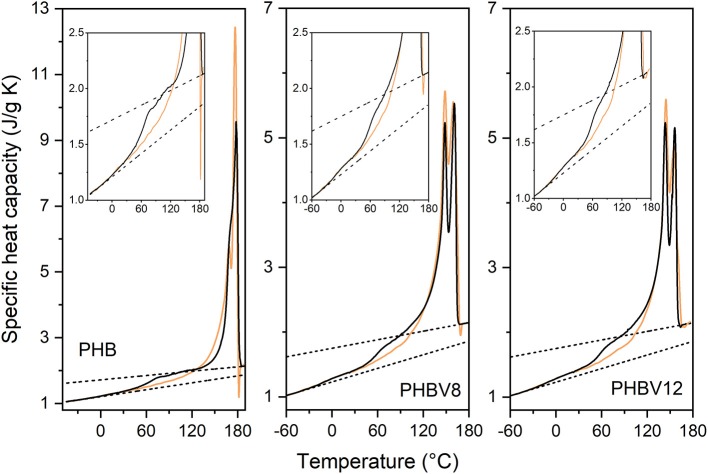
Apparent specific heat capacity (*c*_*p,app*_ at the heating rate of 10 K/min: black solid lines) and reversing specific heat capacity (*c*_*p,rev*_ at the average heating rate of 2 K/min: orange solid lines) of PHB, PHBV8, and PHBV12 as a function of temperature, after storage at *T*_*room*_ for 20 months. The dashed lines are the liquid and solid specific heat capacities ($c_{p}^{s}$ and $c_{p}^{l}$).

The apparent specific heat capacity (*c*_*p,app*_) curves of PHB, PHBV8, and PHBV12, after processing at 80°C for 4 min and storage at *T*_*room*_ for increasing *t*_*a*_s are reported in Figures 2–4. The figures display that Δ*c*_*p*_ at the respective *T*_*g*_s decreases with increasing *t*_*a*_, which proves progressive and further solidification of the samples during the aging. In parallel, a *c*_*p,app*_ increase is observed at temperatures higher than about 40°C for all the samples. For PHB, this *c*_*p,app*_ increase has been mainly associated to a process of enthalpy recovery subsequent to structural relaxation of the RAF, and thus connected to the RAF devitrification (Di Lorenzo et al., 2012). A clear demonstration that the endothermic process at temperatures slightly higher than the respective *T*_*g*_s is an irreversible event is provided by Figure 5, which shows a comparison between the *c*_*p,app*_ curves and the *c*_*p,rev*_ curves of PHB, PHBV8, and PHB12 after aging at *T*_*room*_ for 20 months. The *c*_*p,app*_ and *c*_*p,rev*_ curves overlap in the *T*_*g*_ region, centered approximately at 0°C for PHB, and −10°C for PHBV8 and PHBV12, but strongly diverge at temperatures higher than about 40°C. This attests that the process occurring at temperatures higher than 40°C is an event that does not follow the temperature modulation, and proceeds irreversibly during the heating run. It could be an irreversible melting, and/or an enthalpy recovery following the RAF structural relaxation. For PHB it was demonstrated quantitatively that this endothermic process cannot be connected only to irreversible melting, because it would involve a small portion of the crystalline phase, whereas the simultaneous *c*_*p,rev*_ increase in the temperature range 40–70°C corresponds to the mobilization of a high percentage of chains. This holds also for PHBV8 and PHBV12: in the present case, for PHB the *c*_*p,rev*_ increase in the temperature range 40–70°C correspond to the mobilization of about 20% of the sample, whereas for PHBV8 and PHBV12, in the temperature range 40–60°C, to the mobilization of about 15% of the sample. Thus, enthalpy recovery following the RAF structural relaxation mainly contributes to the endothermic event starting at about 40°C, although also fusion of imperfect crystals grown during storage at *T*_*room*_ can simultaneously occur. At higher temperatures, the crossing of the *c*_*p,app*_ and *c*_*p,rev*_ curves for all the samples proves the occurring of an intense reversing melting, which involves melting and concomitant recrystallization during scans at relatively low heating rate.

Due to the presence of the RAF endothermic enthalpy recovery process, the exact beginning of the fusion is unknown, so that it is impossible to calculate correctly the crystallinity degree from the *c*_*p,app*_ curves, even if the *c*_*p,rev*_ curves are used as thermodynamic (baseline) specific heat capacity at temperatures slightly above *T*_*g*_ (Di Lorenzo et al., 2012). In addition, it has to be taken into account that the temperature dependence of the enthalpy of fusion of 100% crystalline PHBV8 and PHBV12 is unknown, and likely different from that of the homopolymer PHB (Righetti et al., 2013). Therefore, the estimation of the crystalline fractions of the PHB, PHBV8, and PHB12 samples after aging at *T*_*room*_ for different *t*_*a*_s was performed by XRD analysis.

### XRD Analysis of PHB, PHBV8, and PHBV12 During Aging at *T_room_*

**Figure 6:**
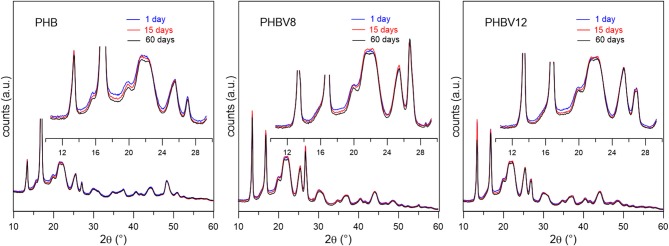
Instrumental-background corrected XRD patterns of PHB, PHBV8, and PHBV12 after storage at *T*_*room*_ for select aging times *t*_*a*_s. The bottom sections are the entire collected XRD patterns, whereas the top sections are enlargements in the 2θ range in which the most intense reflections are observed.

Figure 6 collects selected XRD scans of PHB, PHBV8, and PHBV12 after different aging times at *T*_*room*_. The XRD profiles are in perfect agreement with previous reported data (Scandola et al., 1992; Wang et al., 2001). The starting samples are highly crystalline, but a further increase in crystallinity with aging time, although small, is real. In the insets of Figure 6, both the small increase in peak intensities and the reduction of the amorphous halo, which is numerically relevant since extends in a broad angular range, are appreciable. The XRD profiles show the presence of some crystal orientation in the as-prepared samples, as often occurs in injection molded samples (Hsiao et al., 2013), which however does not change during the storage at *T*_*room*_. The calculated crystal fraction (*X*_*C*_) values after different aging times at *T*_*room*_ are reported and discussed in the following section.

From the position of the most intense peaks, the crystallographic parameters were derived, as reported in Table 4. As a consequence of the increase in HV co-monomer content, which is characterized by higher steric dimensions, the lattice cell units of the copolymers, as well as the cell volume, slightly increase with respect to those of PHB, which proves that the HV units are partially included in the PHB lattice, although it is impossible to derive its percentage. As the difference in HV content in the samples PHBV8 and PHBV12 is small, the relative crystallographic parameters are similar.

**Table 4:** Crystallographic parameters of PHB, PHBV8, and PHBV12.

	***a* (Å)**	***b* (Å)**	***c* (Å)**	***V* (Å^**3**^)**
PHB	5.69 ± 0.02	13.18 ± 0.03	5.89 ± 0.03	442 ± 4
PHBV8	5.70 ± 0.02	13.23 ± 0.05	5.93 ± 0.06	447 ± 7
PHBV12	5.72 ± 0.02	13.22 ± 0.05	5.94 ± 0.06	449 ± 7

From the cell parameters, by assuming, according to previous studies, that approximately half of the HV co-units are included in the PHB lattice (Kamiya et al., 1991; Bonthrone et al., 1992), the density values of 1.287 and 1.285 g/cm^3^ were calculated for the totally crystalline PHBV8 and PHBV12 copolymers, respectively.

### Evolution of the Crystalline, Mobile Amorphous, and Rigid Amorphous Weight Fractions of PHB, PHBV8, and PHBV12 During Aging at *T_room_*

From the *c*_*p,app*_ curves (Figures 2–4), the mobile amorphous weight fractions at 25°C, after aging at *T*_*room*_ for different *t*_*a*_s, were calculated for PHB, PHBV8, and PHBV12, as *X*_*MAF*_ = Δ*c*_*p*_ (25°C)/Δ$c_{p}^{a}$ (25°C), where Δ$c_{p}^{a}$ is the specific heat capacity increment of the totally amorphous samples (Δ$c_{p}^{a}$ = $c_{p}^{l}$ – $c_{p}^{s}$). The crystalline weight fractions were assumed equal to the crystal fractions obtained by XRD analysis, and the rigid amorphous weight fractions (*X*_*RAF*_) were deduced by difference (*X*_*RAF*_ = 1 – *X*_*C*_ – *X*_*MAF*_).

**Figure 7:**
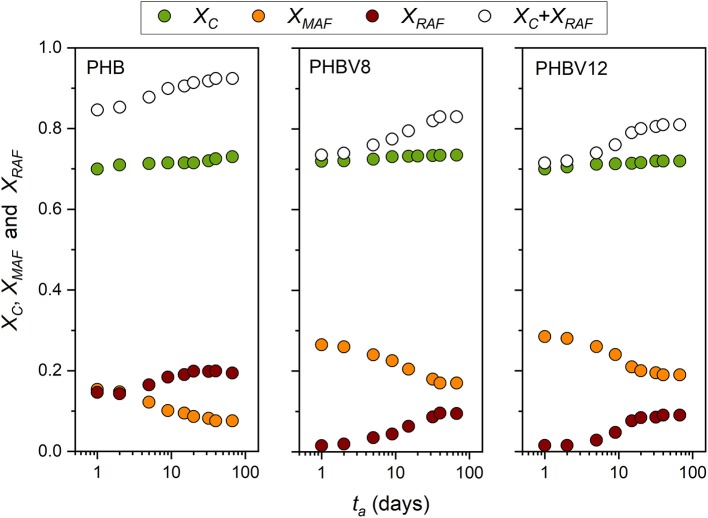
Time evolution of the crystalline weight fraction (*X*_*C*_), mobile amorphous weight fraction (X_*MAF*_), rigid amorphous weight fraction (*X*_*RAF*_), and total solid fraction (*X*_*C*_+ *X*_*RAF*_) of PHB, PHBV8 and PHBV12 as a function of the storage time (*t*_*a*_) at *T*_*room*_ (estimated errors: ±0.02 for *X*_*C*_ and *X*_*MAF*_, ±0.04 for *X*_*RAF*_).

Figure 7 exhibits the evolution of *X*_*C*_, *X*_*MAF*_, and *X*_*RAF*_ for PHB, PHBV8, and PHBV12 as a function of the aging time at *T*_*room*_. The initial crystallinity degree is similar for all the samples. Due to the different molar masses, a quantitative comparison of *X*_*C*_, *X*_*MAF*_, and *X*_*RAF*_ between the homopolymer and the copolymers is not significant. In general, the crystallinity degree increases with reducing the molar mass for fixed crystallization conditions, whereas the RAF content decreases (Androsch, 2008). However, RAF content not lower that 10% have been reported for PHB samples with molar mass of 5,000 g/mol and crystallinity degree ranging from 20 and 80%, which means that RAF in PHB is not negligible.

The initial RAF content in the copolymers is very low. As discussed above, the HV co-units, which are characterized by a higher mobility with respect to the HB units (Laycock et al., 2014), are partially excluded from the crystals, and consequently accrue at the crystal/amorphous interphase. Thus, the mobility of this region turns out to be higher with respect to the homopolymer, and the stiffness reduced.

For all the samples, the crystallinity slightly increases during storage. In parallel *X*_*MAF*_ decreases markedly, which leads to a relevant increase in the RAF. The RAF formation during aging has to be ascribed to the growth of secondary crystals in geometrically restricted areas. In these conditions, the arrangement of the polymeric segments into ordered crystal structures is prevented by the limited chain mobility, which leads to large connection between the crystalline and amorphous areas, with a high number of chain segments subjected to geometrical restrictions.

### Mechanical Properties of PHBV8 and PHBV12 During Aging at *T_room_*

**Figure 8:**
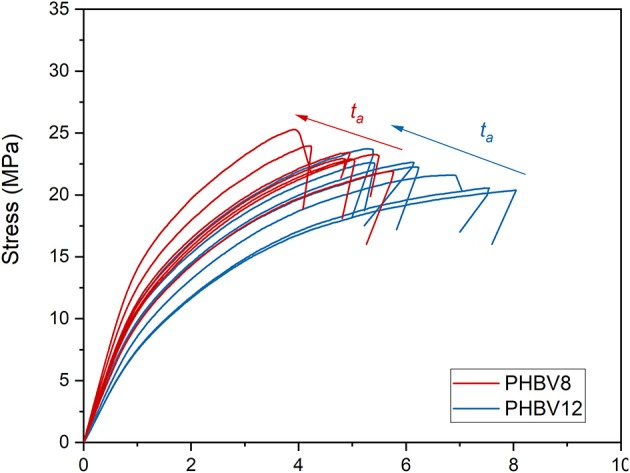
Stress-strain curves for PHBV8 and PHBV12 after different *t*_*a*_s at *T*_*room*_.

The stress/strain curves for PHBV8 and PHBV12 after different *t*_*a*_s at *T*_*room*_ are shown in Figure 8, whereas the values of the elastic modulus (*E*), tensile strength (*TS*), and elongation at break of PHBV8 and PHBV12 are listed in Table 5. For PHB this characterization was possible only after 1 day of aging at *T*_*room*_ (*E* = 3.2 GPa, *TS* = 31.6 MPa, Elongation at break = 1.1%), due to the high stiffness and very low ductility of the homopolymer.

**Table 5:** Elastic modulus (*E*), Tensile strength (*TS*), and Elongation at break of PHBV8 and PHBV12 measured after aging at *T*_*room*_ for different *t*_a_s (estimated errors: ±0.08 GPa for E; ±0.7 MPa for TS; ±0.6% for Elongation at break).

	**PHBV8**			**PHB12**		
***t_***a***_* (days)**	***E* (GPa)**	***TS* (MPa)**	**Elongation at break (%)**	***E* (GPa)**	***TS* (MPa)**	**Elongation at break (%)**
1	1.07	20.8	5.5	0.82	19.5	7.9
3	1.17	21.0	5.0	0.91	19.8	7.5
7	1.23	21.7	4.9	0.99	20.3	6.9
10	1.29	22.3	4.7	1.12	22.0	6.4
15	1.34	23.5	4.9	1.14	22.4	6.2
30	1.52	24.2	4.2	1.26	22.6	5.4
60	1.63	25.1	4.0	1.30	23.4	5.2

**Figure 9:**
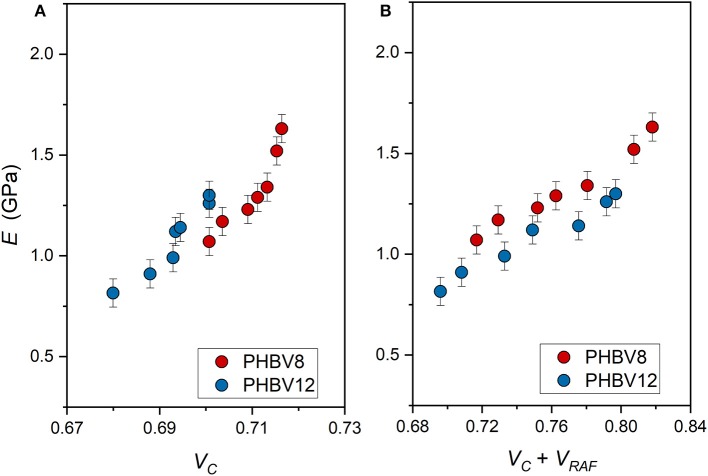
Elastic modulus (*E*) of PHBV8 and PHBV12 after different storage times at *T*_*room*_, as a function of the crystalline volume fraction *V*_*C*_ **(A)** and the total solid volume fraction (*V*_*C*_ + *V*_*RAF*_) **(B)**. The bars are the estimated errors.

The elastic modulus of PHBV8 and PHBV12 is plotted in Figure 9 as a function of: (i) the crystalline volume fraction *V*_*C*_ (Figure 9A) and (ii) the total solid volume fraction (*V*_*C*_ + *V*_*RAF*_) (Figure 9B). The crystalline and rigid amorphous volume fractions, *V*_*C*_ and *V*_*RAF*_, were calculated from the measured *X*_*C*_, *X*_*MAF*_, and *X*_*RAF*_ data, by assuming as density of the crystalline phase, the values reported above (1.287 and 1.285 g/cm^3^, for PHBV8 and PHBV12, respectively), and as the densities of the mobile amorphous phase, the values 1.172 and 1.170 for PHBV8 and PHBV12, respectively (Barker et al., 1990). The density of the RAF was assumed equal to that of the amorphous phase, although it is known that the free volume of the RAF is slightly higher than that of the MAF (Lin et al., 2002). However, lower density values for the RAF (approximately −10%) did not lead to substantially different trends.

*E* is found increasing with the crystallinity degree, as it is generally expected, with values strongly increasing at the highest *V*_*C*_ values. Conversely, if *E* is plotted against the sum (*V*_*C*_+ *V*_*RAF*_), the trends appear more linear. As the rigid amorphous fraction is made of constrained and stiff amorphous segments, it is reasonable that it contributes to the elastic modulus in a way more similar to that of the crystalline phase. Thus, the apparent better correlation of the experimental *E* data with the sum (*V*_*C*_+ *V*_*RAF*_) suggests that at temperatures above *T*_*g*_, the elastic modulus of the RAF might be close to that of the crystalline phase, as already supposed for poly(1-butene) with a similar analysis Di Lorenzo and Righetti (2008). Figure 9B displays that, for all the total solid fractions investigated, the elastic modulus of PHBV12 is lower than that of PHBV8. This behavior can be ascribed to the slightly higher MAF amount and the slightly higher chain mobility that characterizes PHBV12 with respect to PHBV8 (see Figure 7), which leads to assisted bond rotations in the mobile amorphous fraction.

It is worth noting that, as also discussed above, for PHBV8 and PHBV12, the change in *E* that is detected during storage at *T*_*room*_ has to be ascribed (i) to the progressive increase in crystalline and rigid amorphous fractions, and (ii) to the simultaneous physical aging of the rigid amorphous fraction. The two different processes cannot be separated and distinguished, so that only the resulting effect can be considered and analyzed. Intuitively, the progressive RAF structural relaxation is expected to produce an increased slope of the *E* vs. (*V*_*C*_+ *V*_*RAF*_) trends.

**Figure 10:**
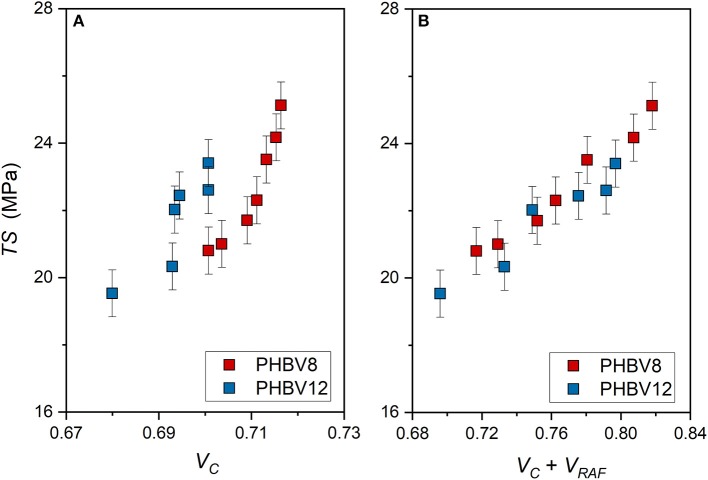
Tensile strength (*TS*) of PHBV8 and PHBV12 after different storage times at *T*_*room*_, as a function of the crystalline volume fraction *V*_*C*_ **(A)** and the total solid volume fraction (*V*_*C*_ + *V*_*RAF*_) **(B)**. The bars are the estimated errors.

Also the tensile strength (*TS*) is found increasing both with *V*_*C*_ and the total solid fraction (*V*_*C*_+ *V*_*RAF*_) (Figure 10). The increase in *TS* at high *V*_*C*_ values is consistent (Figure 10A). However, as for the elastic modulus, the *TS* vs. (*V*_*C*_+ *V*_*RAF*_) trends appear approximately linear for both PHBV8 and PHBV12 (Figure 10B). Differences between the two copolymers are not appreciable. This result suggests that the tensile strength mainly depends on the rigid portions, which inhibit the chain motions, and are similar in the two copolymers.

**Figure 11:**
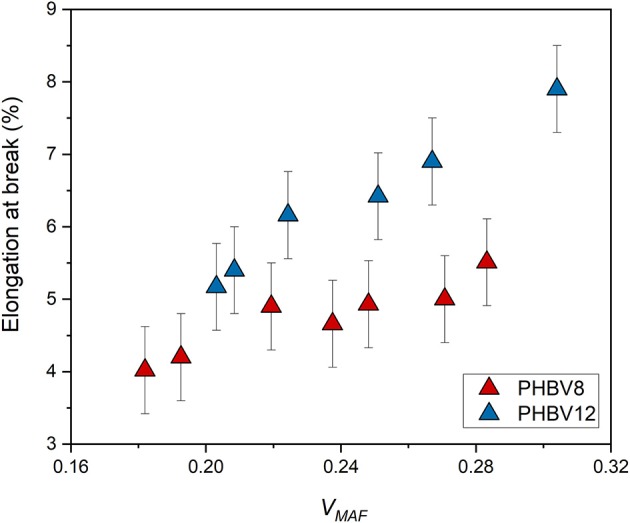
Elongation at break of PHBV8 and PHBV12 after different storage times at *T*_*room*_, as a function of the mobile amorphous volume fraction *V*_*MAF*_. The bars are the estimated errors.

Conversely, the elongation at break is an increasing function of the mobile amorphous volume fraction *V*_*MAF*_ (Figure 11). At high strain, the plastic deformation of semi-crystalline polymers, which involves chain slipping and intermolecular bond breaking, occurs entirely in the amorphous regions. Due to its higher chain mobility, the elongation at break of PHBV12 is higher than that of PHBV8 at a given *V*_*MAF*_ (Figure 11). This behavior is exactly opposite to the one observed for the elastic modulus (see Figure 9).

### Modeling of the Elastic Modulus of PHBV8 and PHBV12

**Figure 12:**
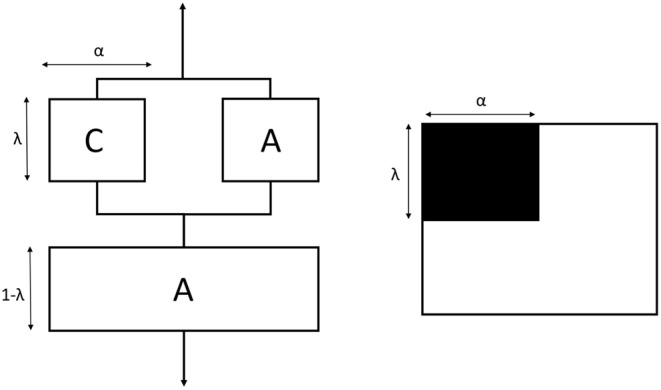
Schematic representation of the classical Takayanagi's two phase model with the equivalent connection in series and parallel. C is the crystalline phase (black) and A is the amorphous phase (white).

In order to estimate the elastic modulus of the crystalline and amorphous phases, the Takayanagi's model was adopted (Takayanagi et al., 1964, 1967). The original Takayanagi's two-phase model represents a mechanical interpretation of the polymer with the two phases (amorphous and crystalline) arranged in series and in parallel. The model was adopted to predict the modulus of the crystalline and amorphous phases, by taking into account that these different parts can undergo a different deformation process under the application of a stress (Prevorsek and Butler, 2006; Ward and Sweeney, 2012; Aliotta et al., 2017). A simple schematization of this model is shown in Figure 12. The parameters λ and α explicate the state of parallel and series coupling of the two phases, with the product λ·α corresponding to the crystalline volume fraction (*V*_*C*_):

(2) $$\begin{array}{l} \lambda =\frac{5V_{C}}{2+3V_{C}} \end{array}$$

(3) $$\begin{array}{l} \alpha =\frac{2+3V_{C}}{5} \end{array}$$

The classical two-phase Takayanagi's expression is the following.

(4) $$\begin{array}{l} \frac{1}{E}=\frac{1-\lambda }{E_{A}}+\frac{\lambda }{\left(1-\alpha \right)E_{A}+\alpha E_{C}} \end{array}$$

where *E*_*A*_ and *E*_*C*_ are the elastic moduli of the amorphous and crystalline phases, respectively, whereas *E* is the elastic modulus of the semi-crystalline material, which can be evaluated from mechanical tests. Numerous applications of the model to blends, composites and nanocomposites can be found in the literature (Cohen and Ramos, 1980; Ji et al., 2002; Loos and Manas-Zloczower, 2013; Zare and Garmabi, 2015).

The classical Takayanagi's two-phase model, if applied to the experimental *E* data listed in Table 5, is found to diverge, leading to unrealistic values. For this simulation, the amorphous fraction was assumed as the sum of the MAF and RAF volumetric fractions. The failure of this classical model to describe the elastic modulus of PHBV8 and PHBV12 was expected: Figure 9A shows that for both PHBV8 and PHV12, at high crystallinity degree, *E* markedly increases when *X*_*C*_ slightly changes. This suggests that different factors have to be taken into consideration in order to correctly interpret and predict the mechanical properties of semi-crystalline polymers.

**Figure 13:**
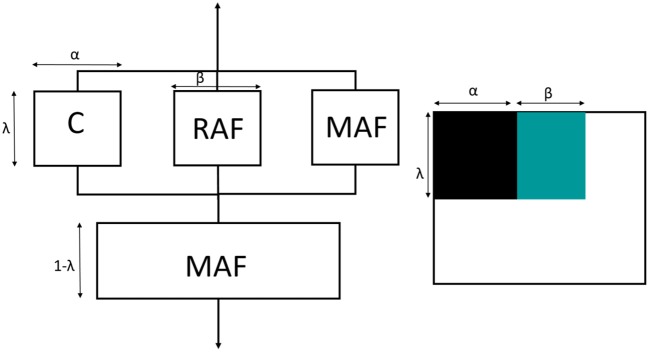
Schematic representation of the modified Takayanagi's three phase model with the equivalent connection in series and parallel. C is the crystalline phase (black), MAF is the mobile amorphous fraction (white) and RAF is the rigid amorphous fraction (green).

Thus, the Takayanagi's model was modified by inserting the rigid amorphous fraction between the MAF and the crystal regions. As for the two-phase model, this cumulative block was arranged in series with the MAF. The graphical representation of the modified Takayanagi's model is showed in Figure 13, whereas the final expression of the inverse of the elastic modulus is given by the following relationship:

(5) $$\begin{array}{l} \frac{1}{E}=\frac{1-\lambda }{E_{MAF}}+\frac{\lambda }{\alpha E_{C}+\beta E_{RAF}+\left(1-\alpha -\beta \right)E_{MAF}} \end{array}$$

where *E*_*C*_, *E*_*MAF*_, and *E*_*RAF*_ are the moduli of the crystalline, mobile amorphous and rigid amorphous fractions, respectively. As the RAF and the crystal phase are strictly connected, and likely RAF exhibits a mechanical behavior similar to that of the crystal phase, the state of parallel and series coupling for the modified model was slightly changed with respect to Equation (2):

(6) $$\begin{array}{l} \text{λ}=\frac{5(V_{C}+V_{RAF})}{2+3(V_{C}+V_{RAF})} \end{array}$$

where *V*_*C*_, *V*_*MAF*_, and *V*_*RAF*_ are the volumetric fractions of the crystalline, mobile amorphous and rigid amorphous fractions, respectively. Accordingly, the parameters α and β are defined as *V*_*C*_/λ and *V*_*RAF*_/λ, respectively. For the calculation of the volumetric fractions, the density of the RAF was assumed equal to that of the amorphous phase. Lower density values for the RAF (approximately −10%) did not lead to substantially different results.

By assuming:

(7) $$\begin{array}{l} y=\frac{1}{E} \end{array}$$

(8) $$\begin{array}{l} X_{1}=\frac{1}{E_{MAF}} \end{array}$$

(9) $$\begin{array}{l} X_{2}=\frac{1}{\alpha E_{C}+\beta E_{RAF}+\left(1-\alpha -\beta \right)E_{MAF}} \end{array}$$

**Figure 14:**
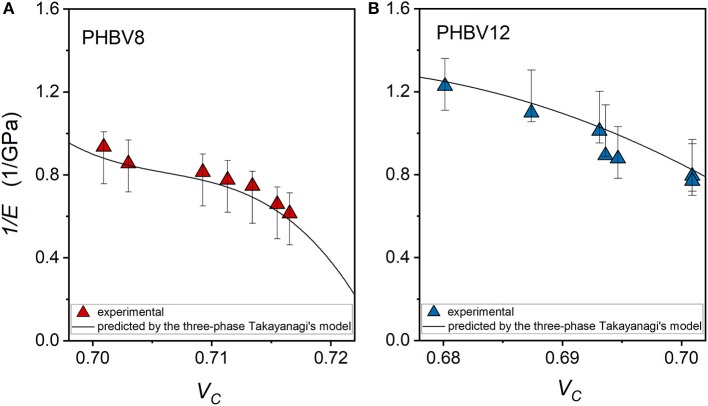
Comparison between the experimental inverse moduli of PHBV8 **(A)** and PHBV12 **(B)** and the inverse values predicted by the modified Takayanagi's model, as a function of the crystalline volume fraction. The bars are the estimated errors.

Equation (5) can be written in a linear form:

(10) $$\begin{array}{l} y=\left(1-\lambda \right)X_{1}+\lambda X_{2} \end{array}$$

which allows the direct determination of (i) *E*_*MAF*_ and (ii) a combination of *E*_*C*_ and *E*_*RAF*_ as a function of the scale parameters α and β, because both the parameters *y* and λ are obtained experimentally. The results of the linear interpolation are reported in Table 6. The comparison between the experimental moduli of PHBV8 and PHB12 and the values predicted by the modified Takayanagi's model, shown in Figure 14, proves that the model describes with good approximation the experimental data.

**Table 6:** Three-phase Takayanagi's model: results of the linear interpolation of Equation (10).

	***E_**MAF**_* (GPa)**	**α*E_***C***_*+ β*E_**RAF**_* (GPa)**
PHBV8	0.18	6.90
PHBV12	0.13	6.78

Due to their high crystallinity, it is impossible to find in the literature experimental data of the elastic modulus of amorphous PHBV copolymers. For a validation of the theoretical *E*_*MAF*_ data listed in Table 6 (average *E*_*MAF*_ = 0.15 GPa), the only useful value that was found refers to a random copolymer with composition: HB (73 mol%) + HV (13 mol%) + HH (14 mol%), where HH means hydroxyheptanoate (Li et al., 2011). The elastic modulus of this totally amorphous copolymer is 0.13 GPa. Although the mobility of the HH co-units is higher than that of HV and HP, a similar order of magnitude guarantees the accuracy of the calculated *E*_*MAF*_ values.

An estimation of the separate *E*_*C*_ and *E*_*RAF*_ values for PHBV8 and PHBV12 was performed with an iterative numerical method (using Excel^®^ Data Solver Function), by maintaining the calculated *E*_*MAF*_ constant, and minimizing the mean square error between the experimental and the theoretical elastic moduli described by Equation (5). The results of the mathematical iteration are reported in Table 7.

**Table 7:** Three-phase Takayanagi's model: results of the mean square error minimization between the experimental and the elastic moduli predicted by Equation (5).

	***E_**RAF**_* (GPa)**	***E_**C**_* (GPa)**
PHBV8	2.09	8.49
PHBV12	2.15	8.45

From Table 7, for both PHBV8 and PHBV12, the elastic modulus of the rigid amorphous fraction (*E*_*RAF*_ = 2.1 GPa) appears lower than the modulus of the crystal phase (*E*_*C*_ = 8.5 GPa), in excellent agreement with intuitive expectation. With respect to the crystal phase, higher free volume and weaker intermolecular bonding can favor chain rearrangement under stress in the RAF regions. On the other hand, *E*_*RAF*_ turns out to be higher than *E*_*MAF*_, due to the higher rigidity and stiffness of the constrained amorphous segments.

The elastic modulus of the crystalline phase appears about two order of magnitude higher than that of MAF. To the best of our knowledge, theoretical studies on the crystalline moduli of the homopolymer PHB and the PHBV copolymers have never been performed, therefore a direct comparison with our *E*_*C*_ prediction is impracticable. In general, polymer crystals exhibit the largest modulus in the chain axis direction. For many polyesters, theoretical values of this limiting elastic modulus are of the order 10–10^2^ GPa (Matsuo et al., 1993; Tashiro, 1993; Wasanasuk and Tashiro, 2012; Kurita et al., 2018; Lee et al., 2018). Due to the strong mechanical anisotropy of polymer crystals, the theoretical crystalline modulus along the chain axis is 1–2 order of magnitude higher than those in different directions (Tashiro and Kobayashi, 1996). Thus, it is reasonable that the calculated *E*_*C*_ values for the copolymers PHBV8 and PHBV12 turns out around 10 GPa, by taking into account the different orientations of the real crystals with respect to the load direction.

It is worth noting that a three-phase Takanayagi's model was applied also to predict the elastic modulus of branched polyethylene (Martín et al., 2013). The model was different from the one used in the present study, because both the amorphous phases (RAF and MAF) were coupled in series and in parallel with crystalline phase. In addition, the *E*_*C*_, *E*_*MAF*_, and *E*_*RAF*_ values were not derived from the model, but values taken from the literature were used. This led, according Martín et al. (2013) to unsatisfactory agreement between the experimental and the predicted *E* values.

## Conclusions

In the present study, for the first time the evolution of tensile mechanical properties of different PHBV copolymers (with 8 and 12 mol% of HV co-units) as a function of the storage time at *T*_*room*_, has been investigated in parallel with the quantification of the crystalline, mobile amorphous and rigid amorphous fractions.

The evolution of *X*_*C*_, *X*_*MAF*_, and *X*_*RAF*_ of PHBV8 and PHBV12 was compared to that of the homopolymer. For all the samples, the crystallinity was found to increase slightly during storage. In parallel *X*_*MAF*_ decreased markedly, with the result that a relevant increase in the RAF was detected. The HV co-units, which are characterized by a higher mobility with respect to the HB units, are partially excluded from the crystals, and consequently accumulate at the crystal/amorphous interphase, with the result the RAF content in the copolymers is lower than that of PHB. For all the samples, the RAF formation during aging was ascribed to the growth, although low, of secondary crystals in geometrically restricted areas. In these conditions, the arrangement of the polymeric segments into ordered crystal structures is prevented by the limited chain mobility, which leads to large coupling between the crystalline and amorphous areas.

It has been demonstrated for PHB, PHBV8, and PHBV12 that the storage at *T*_*room*_ leads to (i) a progressive increase in *X*_*C*_ and *X*_*RAF*_, but also to (ii) a simultaneous physical aging of the rigid amorphous fraction. The two different processes cannot be separated and distinguished, so that only the resulting effect on the mechanical properties can be considered and analyzed.

The experimental elastic modulus was found to increase with the crystallinity degree, although with values strongly increasing at the highest *V*_*C*_ values investigated. Conversely, if *E* is plotted against the sum (*V*_*C*_+ *V*_*RAF*_), the trends appear more regular for both PHBV8 and PHBV12. For all the (*V*_*C*_+ *V*_*RAF*_) values investigated, the elastic modulus of PHBV12 is lower than that of PHBV8, due to the slightly higher MAF amount and the slightly higher chain mobility that characterizes PHBV12 with respect to PHBV8. The tensile strength as a function of (*V*_*C*_ + *V*_*RAF*_) displays an approximate linear trend, with differences between the two copolymers not appreciable. Conversely, the elongation at break turned out to be an increasing function of the mobile amorphous fraction, because at high strain, the plastic deformation of semi-crystalline polymers occurs entirely in the amorphous regions.

In order to estimate the elastic modulus of the crystalline, mobile amorphous and rigid amorphous fractions, the two-phase Takayanagi's model was modified by inserting the rigid amorphous fraction between the MAF and the crystal regions. The mathematical resolution of the model allowed the direct determination of the moduli of the crystalline, mobile amorphous and rigid amorphous fractions for both PHBV8 and PHBV12. The elastic moduli turned out to be quantitatively in the order: *E*_*MAF*_ < *E*_*RAF*_ < *E*_*C*_. The orders of magnitude of all the calculated *E* values appears reasonable and in agreement with experimental results and theoretical expectations.

## Data Availability Statement

All datasets generated for this study are included in the article/supplementary material.

## Author Contributions

MR designed and supervised the study, performed the calorimetric characterization, and wrote the paper. LA performed the modeling of the mechanical properties. NM performed the mechanical measurements. MG performed the XRD characterization. EP performed the molar mass characterization. PC contributed to the design of the study, supervised the sample preparation, and the mechanical characterization. AL contributed to the modeling of the mechanical properties. All the authors reviewed the paper.

### Conflict of Interest

The authors declare that the research was conducted in the absence of any commercial or financial relationships that could be construed as a potential conflict of interest.

## References

[B1] Aliotta L. Cinelli P. Coltelli M. B. Righetti M. C. Gazzano M. Lazzeri A. (2017). Effect of nucleating agents on crystallinity and properties of poly (lactic acid) (PLA). Eur. Polym. J. 93, 822–832 10.1016/j.eurpolymj.2017.04.041

[B2] Androsch R. (2008). Surface structure of folded-chain crystals of poly(R-3-hydroxybutyrate) of different chain length. Polymer 49, 4673–4679. 10.1016/j.polymer.2008.08.026

[B3] Androsch R. Moon I. Kreitmeier S. Wunderlich B. (2000). Determination of heat capacity with a sawtooth-type, power compensated temperature modulated DSC. Thermochim. Acta 357–358, 267–278. 10.1016/S0040-6031(00)00397-X

[B4] Auclerc M. Tauleigne A. Da Cruz Boisson F. Vanhille Bergeron A. Garois N. Fulchiron R. (2019). Polyamide-6 structuration induced by a chemical reaction with a polyether triamide in the molten state. Polymer 172, 339–354. 10.1016/j.polymer.2019.03.033

[B5] Badia J. D. Stromberg E. Karlsson S. Ribes-Greus A. (2012). The role of the crystalline, mobile amorphous and rigid amorphous fractions in the performance of recycled poly(ethylene terephthalate) (PET). Polym. Degrad. Stab. 97, 98–107. 10.1016/j.polymdegradstab.2011.10.008

[B6] Barham P. J. (1984). Nucleation behaviour of poly-3-hydroxy-butyrate, J. Mater. Sci. 19, 3826–3834. 10.1007/BF00980744

[B7] Barham P. J. Keller A. (1986). Relationship between microstructure and mode of fracture in polyhydroxybutyrate. J. Polym. Sci. Part B Polym. Phys. 24, 69–77. 10.1002/polb.1986.180240108

[B8] Barker P. A. Mason F. Barham P. J. (1990). Density and crystallinity of poly(3-hydroxybutyrate/3-hydroxyvalerate). J. Mater. Sci. 25, 1952–1956. 10.1007/BF01045748

[B9] Basset D. C. Flores A. Balta F. J. (1999). Microhardness studies of chain-extended PE: I. Correlations to microstructure. J. Polym. Sci. Part B Polym. Phys. 37, 3151–3158. 10.1002/(SICI)1099-0488(19991101)37:21<3151::AID-POLB24>3.0.CO;2-E

[B10] Bluhm T. L. Hamer G. K. Marchessault R. H. Fyfe C. A. Veregin R. P. (1986). Isodimorphism in Bacterial Poly(β-hydroxybutyrate-co-β-hydroxyvalerate). Macromolecules 19, 2871–2876. 10.1021/ma00165a035

[B11] Bonthrone K. M. Clauss J. Horowitz D. M. Hunter B. K. Sanders J. K. M. (1992). The biological and physical chemistry of polyhydroxyalkanoates as seen by NMR-spectroscopy. FEMS Microbiol. Lett. 103, 269–277. 10.1111/j.1574-6968.1992.tb05848.x

[B12] Bugnicourt E. Cinelli P. Lazzeri A. Alvarez V. (2014). Polyhydroxyalkanoate (PHA): review of synthesis, characteristics, processing and potential applications in packaging. Express Polym. Lett. 8, 791–808. 10.3144/expresspolymlett.2014.82

[B13] Chen Y. Yang G. Chen Q. (2002). Solid-state NMR study on the structure and mobility of the noncrystalline region of poly(3-hydroxybutyrate) and poly(3-hydroxybutyrate-co-3-hydroxyvalerate). Polymer 43, 2095–2099. 10.1016/S0032-3861(01)00792-3

[B14] Cheng M.-L. Sun Y.-M. (2009). Relationship between free volume properties and structure of poly(3-hydroxybutyrate-co-hydroxyvalerate) membranes via various crystallization conditions. Polymer 50, 5298–5307. 10.1016/j.polymer.2009.09.035

[B15] Cheng M.-L. Sun Y.-M. Chen H. Jean Y. C. (2009). Change of structure and free volume properties of semi-crystalline poly(3-hydroxybutyrate-co-hydroxyvalerate) during thermal treatments by positron annihilation lifetime. Polymer 50, 1957–1964. 10.1016/j.polymer.2009.02.025

[B16] Cohen R. E. Ramos R. (1980). Modelling of the viscoelastic behavior of homogeneous and heterogeneous blends of polyisoprene and polybutadiene. J. Macromol. Sci. Part B Phys. 17, 625–651. 10.1080/00222348008212830

[B17] Czerniescka A. Magin A. Schliesser J. Woodfield B. F. Pyda M. (2014). Heat capacity of poly(3-hydroxybutyrate). J. Chem. Thermodyn. 73, 76–84. 10.1016/j.jct.2013.10.020

[B18] de Koning G. J. M. Lemstra P. J. (1993). Crystallization phenomena in bacterial poly[(R)-3-hydroxybutyrate]: 2. Embrittlement and rejuvenation. Polymer 34, 4089–4094. 10.1016/0032-3861(93)90671-V

[B19] Di Lorenzo M. L. Gazzano M. Righetti M. C. (2012). The role of the rigid amorphous fraction on cold crystallization of poly(3-hydroxybutyrate). Macromolecules 45, 5684–5691. 10.1021/ma3010907

[B20] Di Lorenzo M. L. Righetti M. C. (2008). The three-phase structure of isotactic poly(1- butene). Polymer 49, 1323–1331. 10.1016/j.polymer.2008.01.026

[B21] Di Lorenzo M. L. Righetti M. C. (2013a). Effect of thermal history on the evolution of crystal and amorphous fractions of poly[(R)-3-hydroxybutyrate] upon storage at ambient temperature. Eur. Polym. J. 49, 510–517. 10.1016/j.eurpolymj.2012.11.004

[B22] Di Lorenzo M. L. Righetti M. C. (2013b). Evolution of crystal and amorphous fractions of poly[(R)-3-hydroxybutyrate] upon storage. J. Therm. Anal. Calorim. 112, 1439–1446. 10.1007/s10973-012-2734-3

[B23] Doyle M. J. (2000). On the effect of crystallinity on the elastic properties of semicrystalline polyethylene. Polym. Eng. Sci. 40, 330–335. 10.1002/pen.11166

[B24] El-Hadi A. Schnabel R. Straube E. Müller G. Henning S. (2002). Correlation between degree of crystallinity, morphology, glass transition temperature, mechanical properties and biodegradation of poly(3-hydroxyalkanoate) PHAs and their blends. Polym. Test. 21, 665–674. 10.1016/S0142-9418(01)00142-8

[B25] Esposito A. Delpouve N. Causin V. Dhotel A. Delbreilh L. Dargent E. (2016). From a three-phase model to a continuous description of molecular mobility in semicrystalline poly(hydroxybutyrate). Macromolecules 49, 4850–4861 10.1021/acs.macromol.6b00384

[B26] Fatahi S. Ajji A. Lafleur P. G. (2007). Correlation between different microstructural parameters and tensile modulus of various polyethylene blown films. Polym. Eng. Sci. 47, 1430–1440. 10.1002/pen.20836

[B27] Hsiao B. S. Zuo F. Mao Y. (2013). Experimental techniques, in Handbook of Polymer Crystallization, eds Piorkowska E. Rutledge G.C. (Hoboken, NJ: John Wiley & Sons), 1–29.

[B28] Huang J. Ulrich W. Schmauder S. Geiger S. (2011). Micro-mechanical modelling of Young's modulus of semi-crystalline polyamide 6 (PA-6) and elastomer particle-modified-PA 6. Comput. Mater. Sci. 50, 1315–1319. 10.1016/j.commatsci.2010.03.012

[B29] Ji X. L. Jing J. K. Jiang W. Jiang B. Z. (2002). Tensile modulus of polymer nanocomposites. Polym. Eng. Sci. 42, 983–993. 10.1002/pen.11007

[B30] Kamiya N. Sakurai M. Inoue Y. Chûjô R. Doi Y. (1991). Studies of cocrystallization of poly(3-hydroxybutyrate-co-3-hydroxyvalerate) by solid-state high-resolution ^13^C NMR spectroscopy and differential scanning calorimetry. Macromolecules 24, 2178–2182. 10.1021/ma00009a006

[B31] Kawaguchi Y. Doi Y. (1990). Structure of native poly(3-hydroxybutyrate) granules characterized by X-ray diffraction. FEMS Microbiol. Lett. 79, 151–156. 10.1016/S0378-1097(05)80030-8

[B32] Kolesov I. Androsch R. (2012). The rigid amorphous fraction of cold crystallized polyamide 6. Polymer 53, 4770–4777. 10.1016/j.polymer.2012.08.017

[B33] Kunioka M. Doi Y. (1990). Thermal degradation of microbial copolyesters: poly(3-hydroxybutyrate-co-3-hydroxyvalerate) and poly(3-hydroxybutyrate-co-4-hydroxybutyrate). Macromolecules 23, 1933–1936. 10.1021/ma00209a009

[B34] Kuniota M. Tamaki A. Doi Y. (1989). Crystalline and thermal properties of bacterial copolyesters: poly(3-hydroxybutyrate-co-3-hydroxyvalerate) and poly(3-hydroxybutyrate-co-4-hydroxyvalerate). Macromolecules 22, 694–697. 10.1021/ma00192a031

[B35] Kurita T. Fukuda Y. Takahashi M. Sasanuma Y. (2018). Crystalline moduli of polymers, evaluated from density functional theory calculations under periodic boundary conditions. ACS Omega 3, 4824–4835. 10.1021/acsomega.8b00506 31458699 PMC6641976

[B36] Laycock B. Halley P. Pratt S. Werker A. Lant P. (2014). The chemomechanical properties of microbial polyhydroxyalkanoates. Prog. Polym. Sci. 39, 397–442. 10.1016/j.progpolymsci.2013.06.008

[B37] Lee S. Kimoto M. Tanaka M. Tsuji H. Nishino T. (2018). Crystal modulus of poly (lactic acid)s, and their stereocomplex. Polymer 138, 124–131. 10.1016/j.polymer.2018.01.051

[B38] Li S. Y. Dong C. L. Wang S. Y. Ye H. M. Chen G.-Q. (2011). Microbial production of polyhydroxyalkanoate block copolymer by recombinant *Psedomonas putida*. Appl. Microbiol. Biotechnol. 90, 659–669 10.1007/s00253-010-3069-2 21181145

[B39] Lin J. Shenogin S. Nazarenko S. (2002). Oxygen solubility and specific volume of rigid amorphous fraction in semicrystallline poly(ethylene terephthalate). Polymer 43, 4733–4746. 10.1016/S0032-3861(02)00278-1

[B40] Lizundia E. Petisco S. Sarasua J.-R. (2013). Phase-structure and mechanical properties of isothermally melt-and cold-crystallized poly (l-lactide). J. Mech. Behav. Biomed. 17, 242–251. 10.1016/j.jmbbm.2012.09.006 23131793

[B41] Loos M. R. Manas-Zloczower I. (2013). Micromechanical models for carbon nanotube and cellulose nanowhisker reinforced composites. Polym. Eng. Sci. 53, 882–887. 10.1002/pen.23313

[B42] Martín S. Expõsito M. T. Vega J. F. Martínez-Salazar J. (2013). Microstructure and properties of branched polyethylene: application of a three-phase structural model. J. Appl. Polym. Sci. 128, 1871–1878 10.1002/app.38290

[B43] Matsuo M. Sato R. Shimizu Y. (1993). Effect of molecular orientation distribution and crystallinity on the measurement of the crystal lattice modulus of nylon 6 by x-ray diffraction. Colloid Polym. Sci. 271, 11–21. 10.1007/BF00652298

[B44] Nakamura K. Kamiya N. Sakurai M. Inoue Y. Chûjô R. (1992). A molecular mechanics study on conformations of bacterial polyester poly(3-hydroxybutyrate-*co*-3-hydroxyvalerate). Polymer 33, 817–822. 10.1016/0032-3861(92)90342-T

[B45] Nguyen T. L. Bedoui F. Mazeran P.-E. Guigon N. (2015). Mechanical investigation of confined amorphous phase in semicrystalline polymers: case of PET and PLA. Polym. Eng. Sci. 55, 397–405. 10.1002/pen.23896

[B46] Prevorsek D. Butler R. H. (2006). Structure of nylon-6 from analysis of viscoelastic properties. Inter. J. Polym. Mater. Polym. Biomater. 1, 251–277. 10.1080/00914037208075288

[B47] Rastogi R. Vellinga W. P. Rastogi S. Schick C. Meijer H. E. H. (2004). The three-phase structure and mechanical properties of poly(ethylene terephthalate). J. Polym. Sci. Part B Polym. Phys. 42, 2092–2106. 10.1002/polb.20096

[B48] Righetti M. C. Laus M. Di Lorenzo M. L. (2014). Temperature dependence of the rigid amorphous fraction in poly(ethylene terephthalate). Eur. Polym. J. 58, 60–68. 10.1016/j.eurpolymj.2014.06.005

[B49] Righetti M. C. Prevosto D. Tombari E. (2016). Time and temperature evolution of the rigid amorphous fraction and differently constrained amorphous fractions in PLLA. Macromol. Chem. Phys. 217, 2013–2026. 10.1002/macp.201600210

[B50] Righetti M. C. Tombari E. Di Lorenzo M. L. (2013). The role of the crystallization temperature on the nanophase structure evolution of poly[(R)-3-hydroxybutyrate]. J. Phys. Chem. B. 117, 12303–12311. 10.1021/jp4063127 24020615

[B51] Sarge S. M. Hemminger W. Gmelin E. Höhne G. W. H. Cammenga H. K. Eysel W. (1997). Metrologically based procedures or the temperature, heat and heat flow rate calibration of DSC. J. Therm. Anal. 49, 1125–1134. 10.1007/BF0199680211165104

[B52] Scandola M. Ceccorulli G. Pizzoli M. Gazzano M. (1992). Study of the crystal phase and crystallization rate of bacterial poly(3-hydroxybutyrate-co-3-hydroxyvalerate). Macromolecules 25, 1405–1410. 10.1021/ma00031a008

[B53] Schick C. Wurm A. Mohamed A. (2001). Vitrification and devitrification of the rigid amorphous fraction of semicrystalline polymers revealed from frequency-dependent heat capacity. Colloid Polym. Sci. 279, 800–806. 10.1007/s003960100507

[B54] Srubar W. V. III. Wright Z. C. Tsui A. Michel A. T. Billington S. L. Frank C. W. (2012). Characterizing the effects of ambient aging on the mechanical and physical properties of two commercially available bacterial thermoplastics. Polym. Degrad. Stab. 97, 1922–1929: 10.1016/j.polymdegradstab.2012.04.011

[B55] Stern C. Frick A. Weickert G. (2007). Relationship between the structure and mechanical properties of polypropylene: effect of the molecular weight and shear-induced structure. J. Appl. Polym. Sci. 103, 519–533. 10.1002/app.24156

[B56] Sudesh K. Abe H. (2010). Practical Guide to Microbial Polyhydroxyalkanoates. Shrewsbury: Smithers Rapia Technology.

[B57] Sudesh K. Abe H. Doi Y. (2000). Synthesis, structure and properties of polyhydroxyalkanoates: biological polyesters. Progr. Polym. Sci. 25, 1503–1555. 10.1016/S0079-6700(00)00035-6

[B58] Takayanagi M. Imada K. Kajiyama T. (1967). Mechanical properties and fine structure of drawn polymers. J. Polym. Sci. Part C Polym. Symp. 15, 263–281. 10.1002/polc.5070150118

[B59] Takayanagi M. Uemura S. Minami S. (1964). Application of equivalent model method to dynamic rheo-optical properties of crystalline polymer. J. Polym. Sci. Part C Polym. Symp. 5, 113–122. 10.1002/polc.5070050111

[B60] Tashiro K. (1993). Molecular theory of the mechanical properties of crystalline polymers. Prog. Polym. Sci. 18, 377–435. 10.1016/0079-6700(93)90013-3

[B61] Tashiro K. Kobayashi M. (1996). Molecular theoretical study of the intimate relationship between structure and mechanical properties of polymer crystals. Polymer 37, 1775–1786. 10.1016/0032-3861(96)87293-4

[B62] Wang Y. Yamada S. Asakawa N. Yamane T. Yoshie N. Inoue Y. (2001). Comonomer compositional distribution and thermal and morphological characteristics of bacterial poly(3-hydroxybutyrate-co-3-hydroxyvalerate)s with high 3-hydroxyvalerate content. Biomacromolecules 2, 1315–1223. 10.1021/bm010128o 11777409

[B63] Ward I. M. Sweeney J. (2012). Mechanical Properties of Solid Polymers. Chichester: John Wiley & Sons.

[B64] Wasanasuk K. Tashiro K. (2012). Theoretical and experimental evaluation of crystallite moduli of various crystalline forms of poly(l-lactic acid). Macromolecules 45, 7019–7026. 10.1021/ma3010982

[B65] Wunderlich B. (1997). Modeling the heat flow and heat capacity of modulated differential scanning calorimetry. J. Therm. Anal. 48, 207–224. 10.1007/BF01979265

[B66] Wunderlich B. (2003). Reversible crystallization and the rigid amorphous phase in semicrystalline macromolecules. Prog. Polym. Sci. 28, 383–450. 10.1016/S0079-6700(02)00085-0

[B67] Wunderlich B. (2008). Thermal properties of aliphatic nylons and their link to crystal structure and molecular motions. J. Therm. Anal. 93, 7–17. 10.1007/s10973-007-8644-0

[B68] Wurm A. Merzlyakov M. Schick C. (1998). Reversible melting probed by temperature modulated dynamic mechanical and calorimetric measurements. Colloid Polym. Sci. 276, 289–296. 10.1007/s003960050242

[B69] Zare Y. Garmabi H. (2015). A developed model to assume the interphase properties in a ternary polymer nanocomposite reinforced with two nanofillers. Composites Part B 75 29–35 10.1016/j.compositesb.2015.01.031

[B70] Zia Q. Mileva D. Androsch R. (2008). Rigid amorphous fraction in isotactic polypropylene. Macromolecules 41, 8095–8102. 10.1021/ma801455m

